# Superomedial Versus Inferior Pedicle Therapeutic Reduction Mammoplasty for Breast Cancer Patients with 35–40 cm Sternal Notch-to-Nipple Distance

**DOI:** 10.1007/s00266-025-05553-w

**Published:** 2026-01-08

**Authors:** Mohamed F. Asal, Khaled E. Barakat, Ahmed Abdellatif Abdelkader, Ahmed Nagi Abdelaziz, Kaya L. Russell, Ahmed Adham R. Elsayed, Marc D. Basson

**Affiliations:** 1 Surgical Oncology Unit, Surgery Department, Faculty of Medicine, Alexandria University, Alexandria, 21521 Egypt; 2 Borg Al Arab New Hospital, Directorate of Health Affairs in Alexandria, Ministry of Health and Population, Alexandria, 21934 Egypt; 3 College of Medicine, Northeast Ohio Medical University, 4209 St, OH-44, Rootstown, Ohio 44272 USA; 4 Department of Surgery, Northeast Ohio Medical University, Rootstown, Ohio 44272 USA; 5 Department of Biomedical Sciences, Northeast Ohio Medical University, Rootstown, Ohio 44272 USA

**Keywords:** Therapeutic mammoplasty, Superomedial pedicle, Inferior pedicle, Outcomes, Nipple–areolar complex viability

## Abstract

**Background:**

Therapeutic mammoplasty is a well-established option for breast cancer treatment when indicated by oncological evaluation. At sternal notch-to-nipple (SN-N) distances above 35cm, the inferior pedicle (IFP) technique is often considered most reliable due to its vascular supply, potentially reducing complications. However, the superomedial pedicle (SMP) technique is associated with better aesthetic outcomes. We compared SMP and IFP outcomes at SN-N distances of 35–40 cm.

**Methods:**

A retrospective cohort study was conducted, including 81 breast cancer patients (43 SMP, 38 IFP), who underwent therapeutic mammoplasty with an SN-N distance of 35–40 cm. Outcomes compared included operative time, nipple–areola complex (NAC) viability, postoperative complications, and patient satisfaction with breasts, outcomes, and nipples.

**Results:**

The mean SN-N distance was not statistically different between groups. Mean operative time was shorter with SMP than with IFP (88.21 ± 5.07 vs. 119.3 ± 5.60 minutes, *p* < 0.001). NAC viability did not differ significantly, with partial necrosis in 4.7% of SMP patients and 2.6% of IFP patients (*p* = 1.000). Other postoperative complications were rare, with one infection in each group. SMP patients reported higher satisfaction with breasts (88.77 ± 7.23 vs 75.71 ± 8.03, *p* <0.001) and outcomes (90.60 ± 8.19 vs 76.42 ± 8.76, *p* < 0.001). Satisfaction with nipples was high in both groups without a significant difference.

**Conclusion:**

SMP demonstrates comparable or superior outcomes to IFP at SN-N distances of 35–40 cm, expanding the applicability of the superomedial pedicle technique.

**Level of Evidence III:**

This journal requires that authors assign a level of evidence to each article. For a full description of these Evidence-Based Medicine ratings, please refer to the Table of Contents or the online Instructions to Authors www.springer.com/00266.

**Graphical Abstract:**

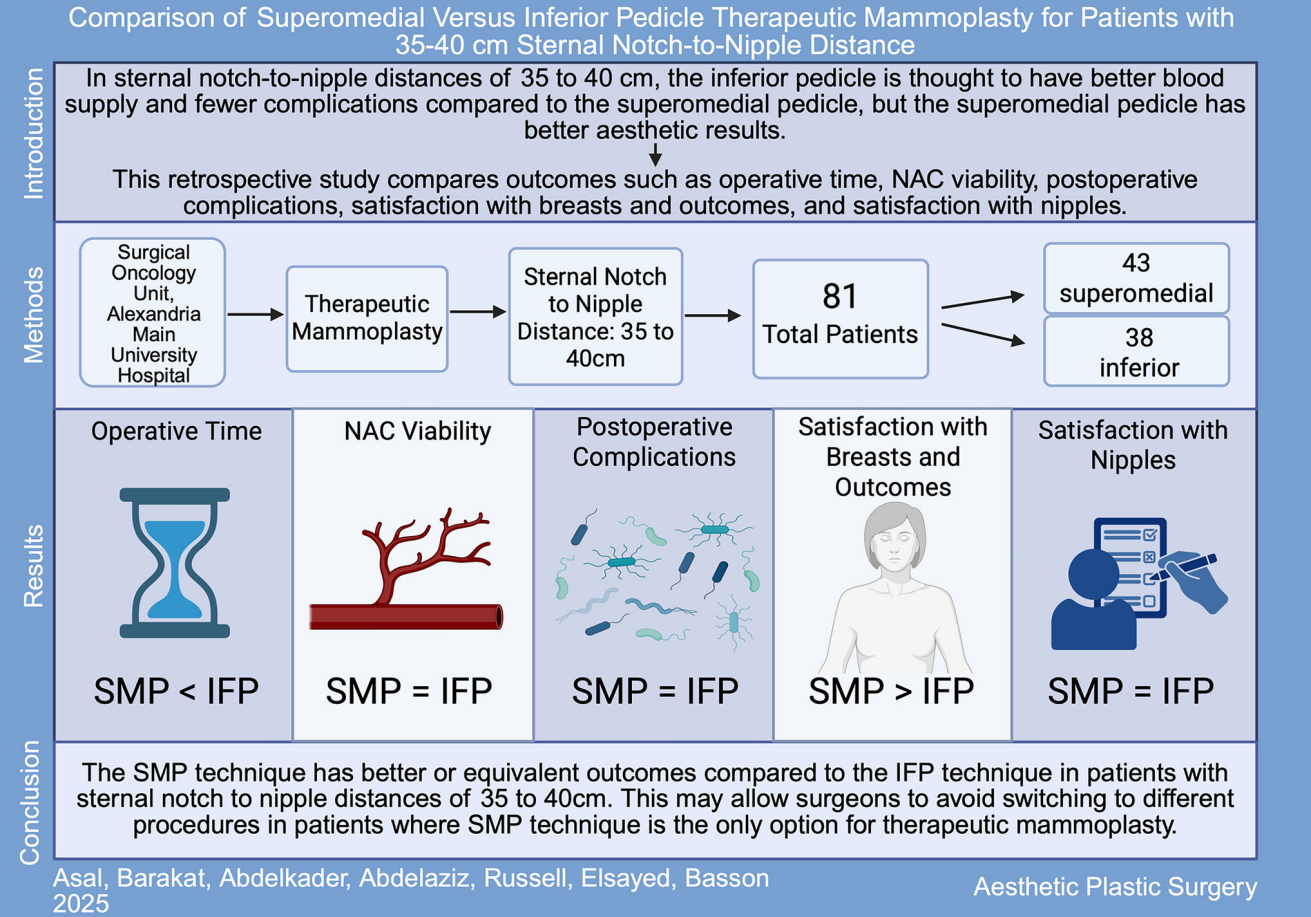

## Introduction

Oncoplastic procedures, such as therapeutic mammoplasty (TM) in specific tumors of the breast in moderate-to-large breasts, provide breast conservation by combining oncological safety, breast reduction, and mastopexy techniques [1]. Breast conservation therapy (BCT) has been the standard of care for breast cancer [1, 2]. Several large cohort studies with long-term follow-up found that BCT and mastectomy had comparable survival rates [3, 4]. Patients receiving BCT exhibit improved disease-free and overall survival [5, 6], as well as an increase in quality of life, when compared to mastectomy [7, 8]. However, in situations where substantial excisions of breast tissues were necessary, unsatisfactory aesthetic results have been reported [9, 10], reducing the use of conventional BCT. Furthermore, BCT has limited uses in individuals with multifocal or multicentric conditions, which are recently managed by oncoplastic procedures such as TM [11–15].

Oncoplastic breast surgery (OBS) was initially introduced by Audretsch in the 1990s when he described a procedure that combines oncologic breast excision with breast reconstruction utilizing plastic surgery [15–17]. OBS techniques that involve partial breast reconstruction are categorized as volume replacement or displacement techniques [9, 18]. TM is a widely used volume displacement technique for women with moderate-to-large breasts and ptosis, allowing concurrent breast reduction and mastopexy while ensuring oncological safety [19–21]. When indicated, contralateral symmetrization can also be performed to optimize aesthetic outcomes [22].

Women with large breasts often experience both physical and psychological discomfort, including back pain, skin irritation under the breasts, depression, and reduced self-confidence [23]. The goal of TM is to remove the tumor with clear margins, restoring the breast’s natural shape by removing unnecessary skin and adipoglandular tissue, while leaving the nipple–areola complex (NAC) properly vascularized. These procedures differ primarily in which pedicle the NAC depends on to stay viable. Despite the fact that no technique has been proven to be the best in the literature, the inferior pedicle-based (IFP) technique is still by far the most commonly performed by American plastic surgeons [24, 25]. The inferior pedicle technique is considered to provide reliable vascular support to the nipple–areola complex, allowing for the safe removal of substantial redundant tissue while reducing the risk of skin flap or NAC necrosis, and can be performed safely even with varying breast pedicle lengths [26].

In recent years, there has been an increase in interest in the superomedial-based pedicle (SMP) mammoplasty approach in both the literature and clinical practice. Evidence suggests that this technique may result in a better cosmetic outcome with less bottoming out and more medial breast fullness over time [27, 28], a shorter operative time [29], but a slightly increased risk of NAC necrosis [30], particularly in cases where the distance between the sternal notch and nipple is equal to or greater than 35 cm. This study aims to compare the outcomes of superomedial (SMP) and inferior pedicle (IFP) techniques in therapeutic mammoplasty in patients with a distance between the sternal notch and the nipple ranging from 35 to 40 cm by evaluating nipple–areola complex (NAC) viability, aesthetic outcomes, operative parameters, postoperative complications, and oncological safety to determine the optimal technique for this specific patient population.

## Methods

### Study Design and Setting

This retrospective cohort study was conducted at the Surgical Oncology Unit, Surgery Department, Alexandria Main University Hospital, between January 1, 2021, and December 31, 2023. The study aimed to compare the clinical outcomes of superomedial pedicle (SMP) and inferior pedicle (IFP) techniques in therapeutic reduction mammoplasty for patients with breast cancer and a sternal notch-to-nipple distance ranging from 35 to 40 cm. This study was approved by the institutional review boards of Alexandria Faculty of Medicine and Northeast Ohio Medical University.

### Patient Selection and Criteria

Patients were included in the study if they had a histologically confirmed diagnosis of breast cancer requiring therapeutic mammoplasty as part of breast-conserving surgery, a preoperative sternal notch-to-nipple distance between 35 and 40 cm, and a C or D cup breast size with sufficient volume for reduction and reshaping. All patients included completed at least one year of follow-up, which encompassed assessment of nipple–areola complex (NAC) viability, Breast *Q* evaluation [31] which was originally reported by Pusic et al, and postoperative complications, all of which were assessed in the clinic by the surgical team. The Breast-*Q* reduction module was used to assess patient-reported outcomes. Breast-*Q* is divided into two primary categories: Quality of Life and Satisfaction. The Quality-of-Life component includes three subscales: psychosocial well-being, sexual well-being, and physical well-being. The Satisfaction category comprises three subscales: satisfaction with breasts, satisfaction with outcome, and satisfaction with nipples. Patients were excluded if they had a sternal notch-to-nipple distance outside the 35–40 cm range, multifocal or multicentric tumors not suitable for breast-conserving surgery, a history of prior breast surgery or radiation therapy affecting the operative field, significant comorbidities or contraindications to surgery (such as poorly controlled diabetes or advanced cardiovascular disease), incomplete medical records or loss to follow-up before full assessment, or if they underwent mastectomy or non-oncoplastic breast procedures.

### Surgical Techniques

The choice of surgical technique was determined primarily by tumor location and size. All surgeons performing the procedures are equally trained in both pedicle techniques, ensuring comparable quality. When tumor location permitted the use of either technique, the choice of pedicle was based on the surgeon’s preference, with the primary objective being to ensure safe oncological margins during tumor removal. Two therapeutic mammoplasty techniques were employed: the superomedial pedicle technique (SMP) and the inferior pedicle technique (IFP). The tumor and surrounding tissue were excised with clear oncological margins confirmed by intraoperative frozen section. The remaining breast tissue was reshaped to achieve an aesthetically pleasing contour, with contralateral symmetrization performed when required.

### Preoperative Assessment

All patients underwent comprehensive preoperative evaluations, including detailed clinical assessment, mammography, ultrasonography, and magnetic resonance imaging (MRI) when indicated. Laboratory investigations included complete blood count, coagulation studies, and renal and liver function tests. Metastatic workup included chest radiography and abdominal ultrasonography for node-negative patients, while computed tomography of the chest, abdomen, and pelvis was performed for node-positive cases. PET-CT was utilized when clinically indicated. Tissue diagnosis was obtained through ultrasound-guided core biopsy, with biological subtype determination including estrogen receptor (ER), progesterone receptor (PR), HER2, and Ki67 status.

### Data Collection

The following variables were collected and analyzed in this study: patient characteristics, including age, body mass index, comorbidities, receipt of neoadjuvant and adjuvant therapies, biological subtype, tumor stage, tumor type, tumor size, axillary status, and tumor location. Surgical outcomes assessed comprised operative time, intraoperative blood loss, length of hospital stay, and specimen weight. Postoperative complications were systematically recorded, specifically noting nipple–areola complex (NAC) necrosis, flap necrosis, hematoma, infection, and wound healing issues. Aesthetic outcomes were evaluated through quality of life and patient satisfaction using the Breast-*Q* questionnaire. Oncological outcomes included analysis of excision margins, local recurrence rates, axillary management, and the requirement for additional adjuvant therapies.

### Statistical Analysis

Data were analyzed using the IBM SPSS software package version 20.0. (Armonk, NY: IBM Corp). Categorical data were represented as numbers and percentages. Chi-square test was applied to compare between two groups. Alternatively, Fisher's exact correction and the Monte Carlo correction test were applied when more than 20% of the cells had an expected count of less than 5. The Chi-square test was applied to investigate the association between the categorical variables. Alternatively, continuous data were tested for normality by the Shapiro–Wilk test. Quantitative data were expressed as range (minimum and maximum), mean, standard deviation, and median. Student’s *t* test was used to compare two groups for normally distributed quantitative variables. On the other hand, the Mann–Whitney test was used to compare two groups for not normally distributed quantitative variables. The significance of the results obtained was judged at the 5% level.

## Results

A total of 81 patients who met the inclusion criteria were divided into two groups based on the surgical technique used: 43 patients underwent the superomedial pedicle (SMP) technique, while 38 patients underwent the inferior pedicle (IFP) technique.

### Demographic and Oncological Characteristics

In this study, there were no statistically significant differences between the two groups regarding sternal notch-to-nipple distance. The mean distance was 37.56 ± 1.69 cm in the SMP group and 37.34 ± 1.73 cm in the IFP group (*p* = 0.572). The patient groups were also comparable, with no significant differences in mean age, marital status, BMI, comorbidities (diabetes, hypertension, or both), or smoking status. Also, there were no significant differences between the two groups in tumor biology, including molecular subtype distribution, neoadjuvant chemotherapy rates, TNM staging, tumor size, axillary lymph node status, tumor side, or tumor location (Table 1).

**Table 1:** Comparison between the two studied groups according to patient demographics and oncological characteristics

	SMP (*n* = 43)	IFP (*n* = 38)	Test of Sig.	*p*
*Sternal notch to nipple (cm)*				
Min.–Max.	35.0–40.0	35.0–40.0	*t*=0.567	0.572
Mean ± SD	37.56 ± 1.69	37.34 ± 1.73		
Median (IQR)	38.0 (36.0–39.0)	37.0 (36.0–39.0)		
*Age (years)*				
Min.–Max.	39.0–70.0	41.0–71.0	*t*=0.541	0.590
Mean ± SD	56.02 ± 7.52	56.92 ± 7.37		
Median (IQR)	57.0 (51.0–61.50)	56.50 (51.0–63.0)		
*Marital status*				
Single	3 (7.0%)	2 (5.3%)	*χ*^2^=1.186	^MC^*p*=0.792
Married	27 (62.8%)	28 (73.7%)		
Divorced	8 (18.6%)	5 (13.2%)		
Widowed	5 (11.6%)	3 (7.9%)		
*BMI (kg/m*^*2*^*)*				
Min.–Max.	25.10–37.10	23.30–37.50	*t*=0.174	0.862
Mean ± SD	30.83 ± 3.46	30.69 ± 3.85		
Median (IQR)	30.40 (28.05–33.60)	30.25 (28.0–33.30)		
*Comorbidities*				
No	28 (65.1%)	23 (60.5%)	*χ*^2^=0.992	^MC^*p*=0.868
DM	6 (14.0%)	8 (21.1%)		
HTN	3 (7.0%)	3 (7.9%)		
HTN+DM	6 (14.0%)	4 (10.5%)		
Smoking	2 (4.7%)	3 (7.9%)	*χ*^2^=0.366	^FE^*p*=0.661
Neoadjuvant therapy	11 (25.6%)	10 (26.3%)	*χ*^2^=0.006	0.940
*Biological subtype*				
Luminal A	20 (46.5%)	19 (50.0%)	*χ*^2^=0.381	0.982
Luminal B	12 (27.9%)	9 (23.7%)		
HER2-Positive	9 (20.9%)	8 (21.1%)		
Triple-Negative	2 (4.7%)	2 (5.3%)		
*Tumor TNM stage*				
IIA	13 (30.2%)	12 (31.6%)	*χ*^2^=0.021	0.990
IIB	21 (48.8%)	18 (47.4%)		
IIIA	9 (20.9%)	8 (21.1%)		
*Tumor size (cm) (max.)*				
Min.–Max.	2.0–5.0	2.0–5.0	*U*=798.000	0.851
Mean ± SD	3.12 ± 1.05	3.16 ± 1.05		
Median (IQR)	3.0 (2.0–4.0)	3.0 (2.0–4.0)		
*Axilla status*				
N0	32 (74.4%)	28 (73.7%)	*χ*^2^=0.285	^MC^*p*=1.000
N1	10 (23.3%)	9 (23.7%)		
N2	1 (2.3%)	1 (2.6%)		
*Side*				
Right	24 (55.8%)	21 (55.3%)	*χ*^2^=0.002	0.960
Left	19 (44.2%)	17 (44.7%)		
*Tumor location (o'clock)*				
Min.–Max.	1.0–11.0	1.0–12.0	*U*=706.500	0.293
Mean ± SD	5.72 ± 2.77	6.63 ± 4.41		
Median (IQR)	6.0 (3.0–8.0)	9.0 (2.0–11.0)		

*IQR* Interquartile range, *SD* Standard deviation, *t* Student *t* test, *U* Mann–Whitney test, *χ*^2^ Chi-square test, *FE* Fisher exact test, *MC* Monte Carlo test, *P* *p*-value for comparing the two studied groups

### Surgical Outcomes and Postoperative Complications

The mean operative time was significantly shorter in the superomedial pedicle (SMP) group (88.21 ± 5.07 minutes) compared to the inferior pedicle (IFP) group (119.3 ± 5.60 minutes) (*p* < 0.001) (Table 2). In contrast, intraoperative blood loss did not differ between the two groups. The SMP group had a mean estimated blood loss of 170.9 ± 24.96 ml, while the IFP group had a mean of 176.3 ± 25.30 ml (*p* = 0.335). Specimen weight was also comparable between the two groups, with a mean of 697.9 ± 51.71 g in the SMP group and 701.6 ± 61.75 g in the IFP group (*p* = 0.772). All patients in both groups were discharged within one day after surgery.

**Table 2:** Comparison between the two groups studied according to surgical outcomes, postoperative complications, and oncological outcomes

	SMP (*n* = 43)	IFP (*n* = 38)	Test of Sig.	*p*
*Operative time (min)*				
Min.–Max.	79.00–99.00	109.0–130.0	*t*=26.190^*^	<0.001^*^
Mean ± SD	88.21 ± 5.07	119.3 ± 5.60		
Median (IQR)	88.00 (84.0–93.0)	119.0 (114.0–123.0)		
*Blood loss (ml)*				
Min.–Max.	150.0–200.0	150.0–200.0	*U*=729.000	0.335
Mean ± SD	170.9 ± 24.96	176.3 ± 25.30		
Median (IQR)	150.0 (150.0–200.0)	200.0 (150.0–200.0)		
*Specimen weight (g)*				
Min.–Max.	590.0–810.0	590.0–810.0	*t*=0.291	0.772
Mean ± SD	697.9 ± 51.71	701.6 ± 61.75		
Median (IQR)	690.0 (665.0–730.0)	710.0 (660.0–750.0)		
*Hospital stay (days)*				
1	(100.0%)	(100.0%)	–	–
*NAC viability*				
No	41 (95.3%)	37 (97.4%)	*χ*^2^=0.231	^FE^*p*=1.000
Partial necrosis	2 (4.7%)	1 (2.6%)		
Flap necrosis	0 (0.0%)	0 (0.0%)	–	–
Hematoma	0 (0.0%)	0 (0.0%)	–	–
Infection	1 (2.3%)	1 (2.6%)	*χ*^2^=0.008	^FE^*p*=1.000
Wound healing issues	0 (0.0%)	0 (0.0%)	–	–
Excision margins	0 (0.0%)	0 (0.0%)	–	–
Recurrence	0 (0.0%)	0 (0.0%)	–	–
Axilla management	0 (0.0%)	0 (0.0%)	–	–
SLNB	32 (74.4%)	28 (73.7%)	*χ*^2^=0.006	0.940
Axillary clearance	11 (25.6%)	10 (26.3%)		
Adjuvant therapy	22 (51.2%)	18 (47.4%)	*χ*^2^=0.116	0.733

*IQR* interquartile range, *SD* standard deviation, *t* Student *t* test, *U* Mann–Whitney test, *χ*^2^ Chi-square test, *FE* Fisher exact test, *P* *p*-value for comparing the two studied groups

*Statistically significant at *p* ≤ 0.05

The viability of the nipple–areola complex (NAC) statistically did not differ significantly between the two groups. Partial NAC necrosis occurred in two patients (4.7%) in the SMP group and in one patient (2.6%) in the IFP group (*p* = 1.000), while no cases of complete necrosis or flap necrosis occurred (Table 2). Other postoperative complications were rare and showed no significant differences between groups. Only one case of surgical site infection occurred in each group (*p* = 1.000). No hematoma or wound healing issues were observed in either group. Both groups achieved negative excision margins in all cases, with no reported local recurrence during the follow-up period. Axillary management was similarly distributed, with sentinel lymph node biopsy (SLNB) performed in 74.4% of SMP cases and 73.7% of IFP cases (*p* = 0.940). Axillary clearance was performed in 25.6% of SMP cases and 26.3% of IFP cases. Adjuvant chemotherapy was administered to 51.2% of the SMP group and 47.4% of the IFP group, with no significant difference noted (*p* = 0.733) (Table 2).

### Patient-Reported Outcomes Using the Breast Q-Reduction Module

At 12 months postoperatively, Breast-*Q* reduction module scores were evaluated to compare between patients who underwent superomedial pedicle (SMP) and inferior pedicle (IFP) therapeutic mammoplasty. Each scale is scored on a scale from 0 (worst) to 100 (best), with higher scores indicating better outcomes and greater patient satisfaction.

Satisfaction with breasts was significantly higher in the SMP group (88.77 ± 7.23) than in the IFP group (75.71 ± 8.03), with a highly statistically significant difference (*p* < 0.001). Similarly, satisfaction with outcome was also significantly higher in the SMP group (90.60 ± 8.19) compared to the IFP group (76.42 ± 8.76), with p < 0.001 (Table 3).

**Table 3:** Comparison between the two groups studied psychosocial well-being, sexual well-being, physical well-being, satisfaction with breasts, and satisfaction with outcomes according to the Breast-Q reduction module at 12 months postoperative

	SMP (*n* = 43)	IFP (*n* = 38)	Test of Sig	*p*
*Psychosocial well-being*				
Min.–Max.	72.0–100.0	72.0–100.0	*t*= 1.865	0.066
Mean ± SD	81.0 ± 7.25	78.50 ± 4.67		
Median (IQR)	78.0 (75.0–84.0)	78.0 (75.0–81.0)		
*Sexual well-being*				
Min.–Max.	65.0–100.0	65.0–100.0	*t*= 0.805	0.424
Mean ± SD	76.89 ± 7.20	75.39 ± 6.57		
Median (IQR)	76.0 (71.0–82.0)	76.0 (71.0–76.0)		
*Physical well-being headaches*				
All of the time (1)	0 (0.0%)	0 (0.0%)	*χ*^2^= 0.322	^FE^*p*=0.701
Some of the time (2)	3 (7.0%)	4 (10.5%)		
None of the time (3)	40 (93.0%)	34 (89.5%)		
*Pain breast*				
All of the time (1)	0 (0.0%)	0 (0.0%)	*χ*^2^= 0.219	^FE^p=0.743
Some of the time (2)	6 (14.0%)	4 (10.5%)		
None of the time (3)	37 (86.0%)	34 (89.5%)		
*Scale score*				
Min.–Max.	82.0–100.0	82.0–100.0	*t*=0.758	0.451
Mean ± SD	93.35 ± 5.45	94.26 ± 5.39		
Median (IQR)	90.0 (90.0–100.0)	90.0 (90.0–100.0)		
*Satisfaction with breasts*				
Min.–Max.	75.0–100.0	66.0–100.0	*t*= 7.702^*^	<0.001^*^
Mean ± SD	88.77 ± 7.23	75.71 ± 8.03		
Median (IQR)	86.0 (82.0–92.0)	75.0 (68.0–82.0)		
*Satisfaction with outcome*				
Min.–Max.	76.0–100.0	68.0–100.0	*t*= 7.525^*^	<0.001^*^
Mean ± SD	90.60 ± 8.19	76.42 ± 8.76		
Median (IQR)	86.0 (86.0–100.0)	76.0 (68.0–86.0)		

*IQR* interquartile range, *SD* standard deviation, *t* Student *t* test, *χ*^2^ Chi-square test, *p* *p*-value for comparing the two studied groups

*Statistically significant at *p*≤ 0.05

In contrast, psychosocial well-being scores were slightly higher in the SMP group (mean ± SD: 81.0 ± 7.25) compared to the IFP group (78.50 ± 4.67), although this difference did not reach statistical significance (*p* = 0.066). Similarly, sexual well-being scores showed no statistically significant difference between the SMP group (76.89 ± 7.20) and the IFP group (75.39 ± 6.57) (*p* = 0.424). Physical well-being scores were comparable between both groups, with a mean of 93.35 ± 5.45 in the SMP group and 94.26 ± 5.39 in the IFP group (*p* = 0.451), indicating no significant difference. Headaches and breast pain are stand-alone items and were minimal in both groups. The majority of patients reported experiencing these symptoms none of the time, and no significant differences were found between the superomedial pedicle (SMP) and inferior pedicle (IFP) groups (*p* > 0.7) (Table 3).

In addition to satisfaction with breast appearance and outcome, patient-reported satisfaction with nipples was evaluated across the five stand-alone items using the Breast-*Q* satisfaction scale. Each item was scored from 1 to 4, where “Very dissatisfied” = 1, “Somewhat dissatisfied” = 2, “Somewhat satisfied” = 3, and “Very satisfied” = 4. Higher scores reflect greater satisfaction.

No statistically significant differences were observed between the SMP and IFP groups across all five nipple-related domains. Mean scores for nipple position were 3.56 ± 0.50 in the SMP group and 3.61 ± 0.50 in the IFP group (*p* = 0.670). Nipple alignment demonstrated excellent symmetry in both groups (SMP: 3.86 ± 0.35; IFP: 3.87 ± 0.34; *p* = 0.917). Satisfaction with the shape of the nipples and areolas did not differ significantly (*p* = 0.160), nor did satisfaction with their appearance (SMP: 3.63 ± 0.49; IFP: 3.76 ± 0.43; *p* = 0.191). Satisfaction with nipple sensation was also comparable between groups (SMP: 3.53 ± 0.55; IFP: 3.50 ± 0.56; *p* = 0.773) (Table 4).

**Table 4:** Comparison between the two groups studied according to satisfaction with nipples

	SMP (*n* = 43)				IFP (*n* = 38)				*χ*^2^	*p*
	Very Dissatisfied	Somewhat Dissatisfied	Somewhat Satisfied	Very Satisfied	Very Dissatisfied	Somewhat Dissatisfied	Somewhat Satisfied	Very Satisfied		
High low	0 (0.0%)	0 (0.0%)	19 (44.2%)	24 (55.8%)	0 (0.0%)	0 (0.0%)	15 (39.5%)	23 (60.5%)	0.184	0.668
Lined up	0 (0.0%)	0 (0.0%)	6 (14.0%)	37 (86.0%)	0 (0.0%)	0 (0.0%)	5 (13.2%)	33 (86.8%)	0.011	0.917
Shape	0 (0.0%)	2 (4.7%)	14 (32.6%)	27 (62.8%)	0 (0.0%)	0 (0.0%)	9 (23.7%)	29 (76.3%)	2.474	^MC^*p*=0.296
Look	0 (0.0%)	0 (0.0%)	16 (37.2%)	27 (62.8%)	0 (0.0%)	0 (0.0%)	9 (23.7%)	29 (76.3%)	1.729	0.188
Sensation	0 (0.0%)	1 (2.3%)	18 (41.9%)	24 (55.8%)	0 (0.0%)	1 (2.6%)	17 (44.7%)	20 (52.6%)	0.347	^MC^*p*=0.908

*χ*^2^ Chi-square test, *MC* Monte Carlo test, *P* *p*-value for comparing the two studied groups

### Discussion

Therapeutic mammoplasty, when consistent with oncologic principles, is an effective treatment option for select patients with breast cancer if there is sufficient breast volume for resizing and reshaping [20]. Although not all patients in this study were eligible for both the superomedial pedicle (SMP) and inferior pedicle (IFP) approaches due to variations in tumor size, position, and oncological evaluation, comparing the outcomes in terms of nipple–areola complex viability, aesthetic outcomes, operative parameters, postoperative complications, and oncological safety between both techniques based on a sternal notch-to-nipple (SN-N) distance of 35 to 40cm can guide clinical practice. Current literature recommends avoiding the SMP approach in patients with an SN-N distance exceeding 30–35cm [30], whereas the IFP approach can be used with greater distances [32]. In situations where only the SMP approach is possible (i.e., a tumor in the inferior pedicle), but the SN-N distance is above 35cm, surgeons may use a different procedure even if it has lower patient satisfaction. This study aimed to compare the outcomes of the SMP technique with the IFP technique in SN-N distances between 35 and 40cm to expand the applicability of the SMP approach when it agrees with the oncological evaluation. We found the mean operative time to be shorter with SMP than with IFP. Nipple–areola complex (NAC) viability was similar. Postoperative complications were minimal and of equivalent frequency in both groups. Patient satisfaction regarding breast appearance and outcome was higher after SMP than IFP. Patient satisfaction with nipples was similar.

The SMP group had significantly shorter operative time than the IFP group because the superomedial pedicle is shorter and requires less dissection, including less flap de-epithelialization, decreased superior flap creation or reshaping, and single en bloc resection of breast tissue [29]. Additionally, the SMP approach tends to have simpler NAC transposition because it is a shorter, more direct pedicle, while the IFP approach requires more trimming, rotation, or shaping of the pedicle to allow for optimal NAC placement [27]. Decreased operative time is desirable for many reasons, including reducing the risk of complications like surgical site infections [33], decreased exposure to anesthesia [34], faster recovery time [35], and increased patient satisfaction.

**Fig. 1:**
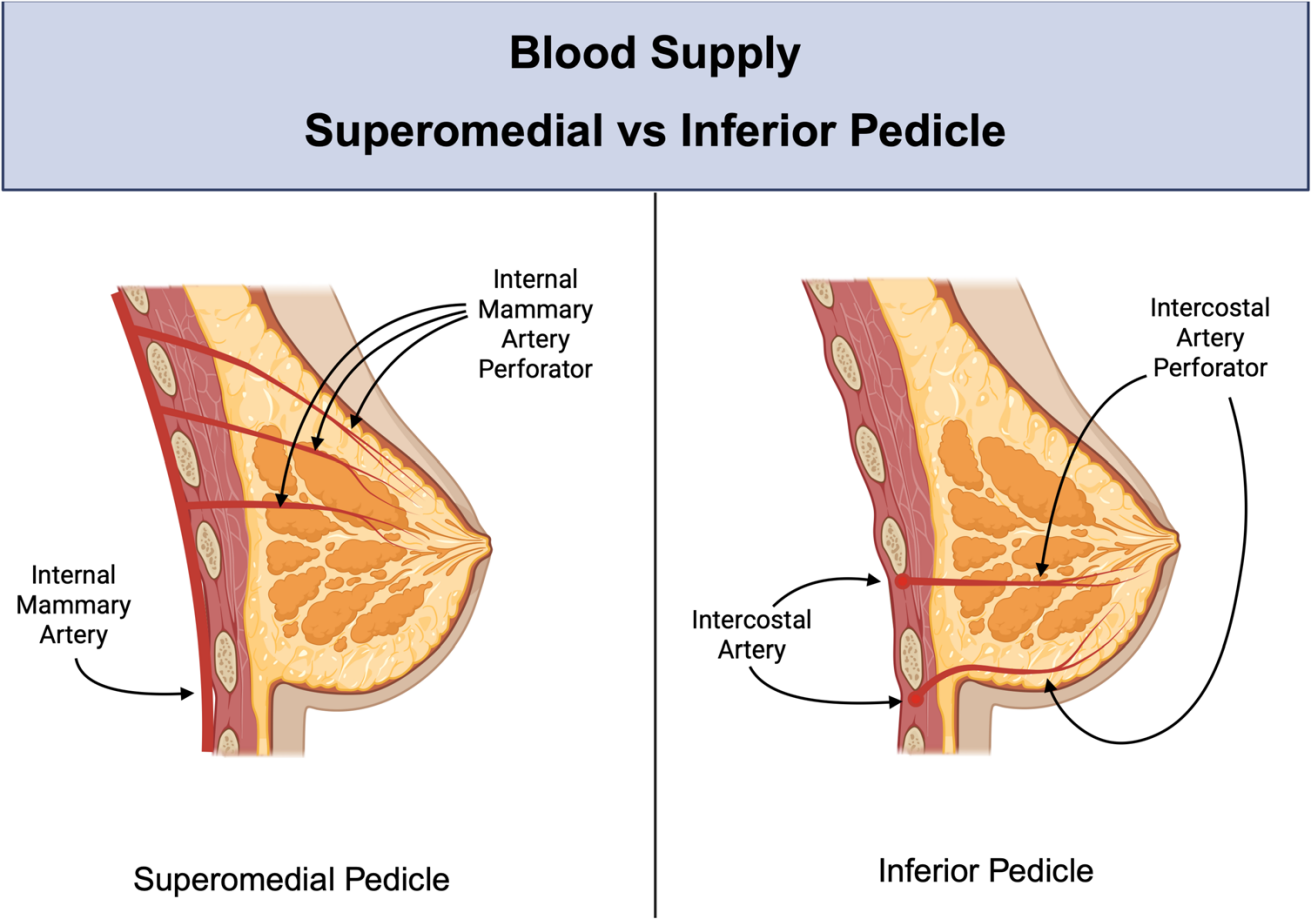
Blood supplies of the superomedial pedicle and inferior pedicle. Created in BioRender. Basson, M. (2025) https://BioRender.com/gh0w6fm

Both the SMP and IFP approaches had similar, effective NAC viability. Currently, the IFP technique is favored by surgeons at SN-N distances between 35 and 40cm because it is believed to have a better blood supply at these distances, which could lead to decreased complication rates relating to the NAC [36] (Fig. 1). The blood supply to the nipple–areola complex (NAC) differs significantly between superomedial and inferior pedicle techniques in breast reduction surgery. The superomedial pedicle relies primarily on perforating branches from the internal mammary artery, particularly from the second and third [37, 38]. These perforators run superficially, approximately 1 cm deep to the skin, traveling radially toward the nipple in the subcutaneous tissue [37]. In contrast, the inferior pedicle technique depends on the fourth, fifth, and sixth intercostal perforators [39, 40]. These perforators emerge from the intercostal arteries, which come from the internal mammary artery, and create an axial pattern blood supply to the NAC [39]. A large musculocutaneous perforator from either the fifth or sixth branch, typically located 2–4 cm above the inframammary fold and medial to the breast meridian, sustains the inferior-based pedicle [38]. Although some studies report increased risk of NAC necrosis when using the SMP technique in SN-N distances above 30 cm [30], the results of this study suggest that the superomedial pedicle has a blood supply capable of maintaining NAC viability even at SN-N distances exceeding 35cm. An important aspect of maintaining sufficient blood supply to the NAC in the SMP technique is preserving the perforators of the internal mammary artery [37]. These perforators are the main blood supply of the NAC from the superomedial pedicle [37]. In cases where the SMP technique is the only viable option based on tumor location, these results give the opportunity to use therapeutic mammoplasty in patients with SN-N distances of 35–40cm rather than switching to a different surgery.

Other postoperative complications were rare in each group, with no significant differences between groups. SMP and IFP groups each reported one case of surgical site infection, resulting in rates of 2.3% and 2.6%, respectively. These values are consistent with the literature, which reports postoperative complication rates associated with therapeutic mammoplasty between 0.6 and 4.6% [41]. This indicates that therapeutic mammoplasty, whether done by SMP or IFP, as long as it doesn’t contradict oncological evaluation, is a safe and effective procedure with low rates of cancer recurrence.

**Fig. 2:**
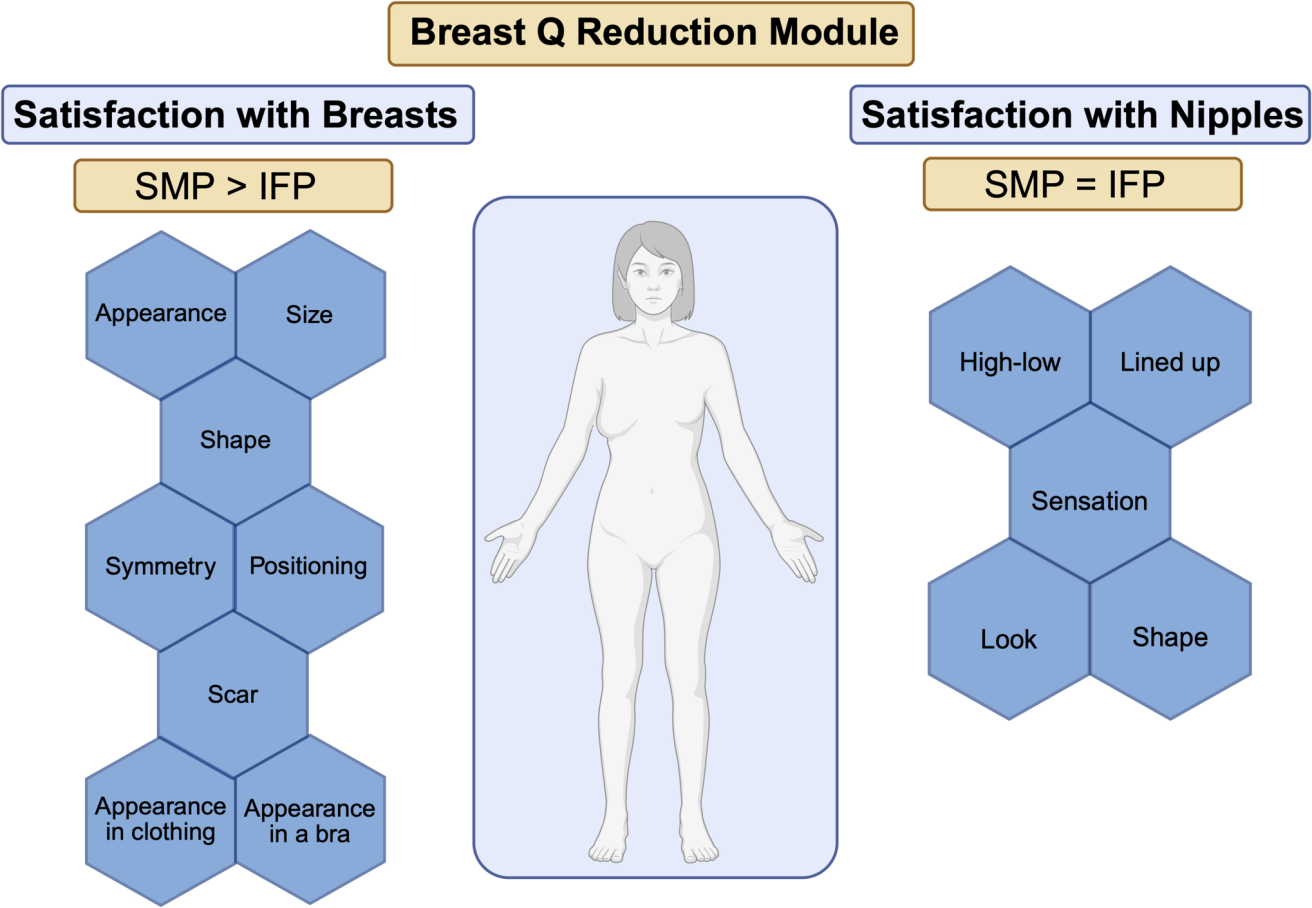
Components of the Breast-Q reduction module: satisfaction with breasts and satisfaction with nipples. Created in BioRender. Basson, M. (2025) https://BioRender.com/l8zy0ik

Compared with the IFP group, the SMP group scored significantly higher in patient satisfaction with breasts and overall surgical outcomes according to the Breast-Q reduction module (Fig. 2). Categories included in this questionnaire include patients’ satisfaction with breast appearance, size, shape, symmetry, bra fit, scar appearance, and positioning of the breasts, as well as how they look in clothing and in the mirror. Satisfaction with overall surgical outcomes measures patients’ overall evaluation of their surgical experience and if the results met their expectations, as well as their sense of decision satisfaction, emotional benefit, and willingness to recommend or repeat the surgery. It has been well established that the superomedial technique typically provides better aesthetic outcomes [27, 28]. This is because the SMP technique typically leads to increased medial fullness by incorporating more medial breast tissue into the pedicle [27]. Additionally, the support provided by the medial pedicle in the SMP technique leads to decreased rates of bottoming out and longer-lasting aesthetic results [42]. Increased medial fullness and decreased bottoming out both lead to more youthful appearing breasts for an extended period of time, which leads to increased patient satisfaction compared to the results of the IFP technique [27]. Patient dissatisfaction with breast appearance can have negative effects on self-confidence, mental health, and overall well-being [42], so it is vital for surgeons to take into consideration the potential aesthetic outcomes of either technique when choosing which one to use.

Both techniques had high patient satisfaction with nipples, with no significant difference between groups. Not only is the SMP technique capable of supplying blood, which would impact patient satisfaction with sensation of nipples, but the SMP technique also scored similarly to the IFP technique in patient satisfaction with nipple height, alignment of nipples, nipple shape, and the appearance of nipples. The SMP technique, having similar nipple aesthetic results to the IFP technique, further supports the idea that the superomedial pedicle has a viable blood supply to the NAC in SN-N distances of 35–40cm. If the NAC did not receive an adequate blood supply, lower scores in all categories would be expected. Much like patient dissatisfaction with breast appearance, patient dissatisfaction with nipples can have negative impacts on several aspects of their lives, such as self-confidence and mental health.

This study is limited by its retrospective design and small sample size, which makes controlling for patient selection challenging. Nevertheless, potential confounding factors such as age, BMI, and other comorbidities did not differ significantly between groups. While all patients and procedures were managed by the same surgeons, outcomes may vary when performed by other surgeons. The two groups were not entirely comparable, since some patients were not eligible for both techniques based on oncological assessment. This introduces a risk of selection bias, although it reflects real-world decision-making, which limits the strength of a direct comparison.

### Conclusion

Therapeutic reduction mammoplasty is a safe and effective treatment option for patients with breast cancer. Oncological evaluation based on tumor size and location is important for determining which pedicle may be used for therapeutic mammoplasty, but current literature does not advise using the superomedial pedicle technique in patients with a sternal notch-to-nipple distance greater than 35cm out of concern that it will lead to inadequate blood supply to the nipple–areola complex [30]. In contrast, this study suggests that at SN-N distances between 35 and 40 cm, the SMP technique had shorter operative times and resulted in higher patient satisfaction with breasts and overall surgical outcomes, with no other significant differences from the IFP technique. When available based on oncological evaluation, the SMP technique can be a viable treatment option in patients with breast cancer with sternal notch-to-nipple distances of 35–40cm, achieving superior aesthetic outcomes while avoiding alternative procedures that are often associated with lower patient satisfaction. An important aspect of maintaining the blood supply of the NAC when using the SMP technique is preserving the perforators of the internal mammary artery, but future research comparing methods of preserving the perforators and how this impacts the blood supply of the NAC in the SMP approach is needed.
